# Rehabilitation Therapy Staffing Composition and Post-Acute Care Quality in Skilled Nursing Facilities

**DOI:** 10.1093/geroni/igaa057.301

**Published:** 2020-12-16

**Authors:** Xiao (Joyce) Wang, Jennifer Hefele, Laura Driscol, Joyce Wang

**Affiliations:** 1 University of Massachusetts, Boston, Massachusetts, United States; 2 Booz Allen Hamilton, Arlington, Virginia, United States; 3 Boston University College of Health and Rehabilitation Sciences: Sargent College, Boston, Massachusetts, United States; 4 University of Massachusetts Boston, Boston, Massachusetts, United States

## Abstract

Rehabilitation therapy staffing is crucial in achieving high quality of post-acute care (PAC) in skilled nursing facilities (SNFs), but few studies have explored therapy staffing composition and how it relates to SNFs’ PAC quality. This study describes SNFs’ therapy staffing composition and its association with facility-level PAC fall rates. Our study was a cross-sectional study using facility-level data (Q3 2017-Q2 2018). Data sources include Nursing Home Compare, Payroll-Based Journal data, Area Health Resource File and LTCFocUS.org. The first independent variable was the share of total therapy staff in the direct care team, a ratio between total therapy staffing hours and total direct care hours (includes nursing).To further understand the composition of therapy staff with different qualifications within the therapy team, two variables were generated: 1) proportion of higher skilled therapy staff (i.e. assistant, therapist) hours of total therapy hours; and 2) assistant to therapist ratio. Multivariate linear regression modeling was used, controlling for other characteristics and state fixed effects. Our results show SNF therapy staffing compositions varied significantly by profit status, chain affiliation, and urban/rural location. Further, SNFs with higher shares of therapy staff and higher skilled staff had significantly lower fall rates. However, SNFs with higher assistant-to-therapist ratios had higher fall rates. Our results demonstrated the value of having a multidisciplinary team with higher skilled staff. The results also supported researchers’ concerns that recent Medicare payment change (i.e. the Patient Driven Payment Model) may negatively impact quality by reinforcing providers’ incentives of reducing rehabilitation staffing and/or using lower-skilled staff.

